# Trajectories of Suicidal Ideation in People Seeking Web-Based Help for Suicidality: Secondary Analysis of a Dutch Randomized Controlled Trial

**DOI:** 10.2196/jmir.5904

**Published:** 2016-06-30

**Authors:** Trine Madsen, Bregje van Spijker, Karen-Inge Karstoft, Merete Nordentoft, Ad JFM Kerkhof

**Affiliations:** ^1^Copenhagen Mental Health CenterCopenhagen University HospitalHellerupDenmark; ^2^National Institute for Mental Health ResearchResearch School of Population HealthThe Australian National UniversityCanberraAustralia; ^3^Research and Knowledge CentreThe Danish Veteran CentreRingstedDenmark; ^4^Department of Clinical, Neuro, and Developmental Psychology and the EMGOInstitute for Health and Care ResearchFaculty of Behavioural and Movement SciencesVrije UniversiteitAmsterdamNetherlands

**Keywords:** suicidal ideation, online self-help, trajectories, latent growth mixture modeling

## Abstract

**Background:**

Suicidal ideation (SI) is a common mental health problem. Variability in intensity of SI over time has been linked to suicidal behavior, yet little is known about the temporal course of SI.

**Objective:**

The primary aim was to identify prototypical trajectories of SI in the general population and, secondarily, to examine whether receiving Web-based self-help for SI, psychiatric symptoms, or sociodemographics predicted membership in the identified SI trajectories.

**Methods:**

We enrolled 236 people, from the general Dutch population seeking Web-based help for SI, in a randomized controlled trial comparing a Web-based self-help for SI group with a control group. We assessed participants at inclusion and at 2, 4, and 6 weeks. The Beck Scale for Suicide Ideation was applied at all assessments and was included in latent growth mixture modeling analysis to empirically identify trajectories.

**Results:**

We identified 4 SI trajectories. The high stable trajectory represented 51.7% (122/236) of participants and was characterized by constant high level of SI. The high decreasing trajectory (50/236, 21.2%) consisted of people with a high baseline SI score followed by a gradual decrease to a very low score. The third trajectory, high increasing (12/236, 5.1%), also had high initial SI score, followed by an increase to the highest level of SI at 6 weeks. The fourth trajectory, low stable (52/236, 22.0%) had a constant low level of SI. Previous attempted suicide and having received Web-based self-help for SI predicted membership in the high decreasing trajectory.

**Conclusions:**

Many adults experience high persisting levels of SI, though results encouragingly indicate that receiving Web-based self-help for SI increased membership in a decreasing trajectory of SI.

## Introduction

Suicidal ideation (SI) is a common mental health problem. The lifetime prevalence of SI in Western countries has been reported to be around 10% [1,2]. For a person with SI, the prevalence of having attempted suicide is as high as 29% [1]. Variability in intensity of SI over time has previously been linked to suicidal behavior [3,4], yet remarkably little is known about the temporal course in adults with SI.

Traditionally, longitudinal studies have reported the prevalence of self-reported SI at several time points or provided the mean change in SI over time for a whole study cohort or in predefined categories based on, for instance, diagnosis [5-8]. While this has provided valuable knowledge, these studies have not examined trends in individual variability of SI. To our knowledge, only a few studies have examined prototypical trajectories of SI based on individual variation in frequency and course of SI over time. These studies have been carried out in certain predefined subsamples, such as psychiatric patient samples [4,9-12] or in teenage samples [13-16]. Hence, little is known about the individual variation of SI in the adult general population over time.

**Figure 1:**
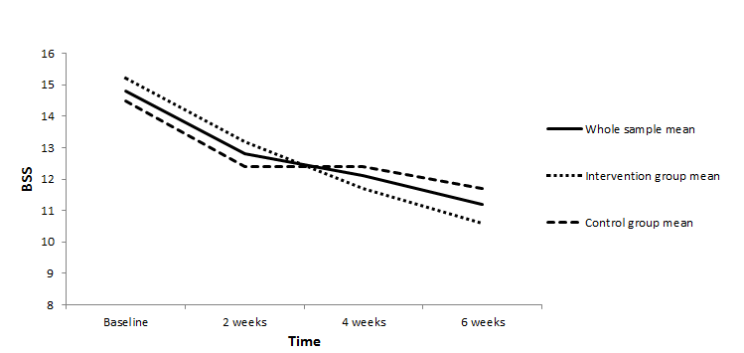
Mean change in suicidal ideation as assessed by the Beck Scale for Suicide Ideation (BSS), overall and by randomization group.

By applying data from a longitudinal Dutch randomized controlled trial comparing a group following a Web-based self-help program for suicidal thoughts with a waitlist control group (Netherlands Trial Register NTR1689), we had the opportunity to examine prototypical patterns of SI in an adult sample from the general population seeking Web-based help for SI. Promising results from this randomized controlled trial have previously been published showing that SI was significantly more reduced in the Web-based self-help program receivers than in the control participants [7]. Yet, while the traditionally applied statistics in that study showed an overall small significant mean effect size of the program (see Figure 1), that study did not evaluate how many trial participants experienced decreasing, increasing, stable, or fluctuating SI over the study period. A better understanding of this individual variation in temporal patterns of SI and what characterizes these patterns may help target preventive actions toward those at greatest risk of suicidal behavior.

Our primary aim was to identify prototypical trajectories of SI, thereby increasing current knowledge on individual variability of SI. Secondarily, we examined whether baseline sociodemographics, clinical symptoms, or the Web-based self-help program was associated with membership in the identified SI trajectories.

## Methods

Full details of the methods of the randomized controlled trial have previously been described [7,17]. Here, we present details of relevance to this study.

### Procedure

We recruited participants from the general population through newspaper advertisements and banners on the Internet that directed interested people to a website where they obtained information about the study and were able to register. Inclusion in the study required a minimum age of 18 years, access to the Internet and an email address, being fluent in Dutch, having mild to moderate SI (defined as a score between 1 and 26 on the Beck Scale for Suicide Ideation, BSS) [18], and not being severely depressed (defined as a score >39 on the Beck Depression Inventory, BDI) [19]. We determined these cutoff scores in consultation with clinical experts. An independent researcher using a block design randomly allocated participants to either the intervention or the control condition.

Because this study was conducted in a vulnerable population, we used safety procedures [7,17]. Each time a participant exceeded a cutoff score on SI (BSS >26) or depressive symptoms (BDI >39), we carried out a risk assessment over the phone. If deemed necessary, or if a participant could not be reached, we contacted their general practitioner. The study was approved by the Medical Ethics Committee of the VU University Medical Centre (registration number 2008/204).

### Intervention

The Web-based self-help program was based on elements from cognitive behavioral therapy, dialectical behavioral therapy, problem-solving therapy, and mindfulness-based cognitive therapy, which have all been shown to reduce suicidality [20-23]. The program consisted of 6 modules for participants to work through independently, ideally doing 1 module per week and spending approximately 30 minutes daily on the module.

Each module contained theory and core exercises. Module 1 aimed at helping participants recognize how often they repeat suicidal thoughts and learning to manage this worrying or ruminating repetition better. Module 2 focused on learning to tolerate and regulate intense emotions. The theory section explained how to recognize an upcoming crisis and tapped into dealing with the urge to self-harm. Core exercises introduced different ways of coping with intense emotions, such as behavioral activation (eg, seeking distraction) and acceptance (waiting until the feelings subside). Participants were also encouraged to make a crisis plan. Modules 3 to 5 dealt with identifying automatic thoughts, recognizing common thinking patterns (all-or-nothing thinking, overgeneralization, or mind reading), and cognitively restructuring the three most important identified negative automatic thoughts. Module 6 was dedicated to preventing relapse and discussed the possibility of future setbacks and disappointments.

The control group received access to a website with information on suicidality, that is, how common it is and its risk factors, and provided a list of common places to seek treatment for suicidality. The control group was provided with access to the self-help program at the 6-week follow-up.

### Measures

Our primary measure, which we used to identify latent trajectories of SI, was the BSS questionnaire, which we distributed to participants at baseline, at weeks 2 and 4 of the intervention, and finally at the sixth and last week of the intervention. Furthermore, we administered questions on the following at baseline, and subsequently applied and examined them as possible predictors of trajectory membership: sex, age, living with partner (yes/no), paid employment (yes/no), having children (yes/no), random allocation group (control/Web-based self-help intervention), and currently receiving other help such as psychiatric or psychological therapy (yes/no). We also examined clinical factors such as having attempted suicide before baseline (item from the BSS), depression symptoms measured by the BDI, and levels of hopelessness as measured by the Beck Hopelessness Scale [24].

### Statistical Methods

We applied latent growth mixture modelling (LGMM) to estimate trajectories of SI. This data-driven statistical method identifies subgroups in a sample based on shared growth parameters (ie, intercept and slope). As such, in LGMM it is assumed that multiple subpopulations exist in a sample; however, no assumptions are made about the number of subpopulations or their specific growth parameters. Hence, individuals are classified into possible unobserved subgroups based on common profile patterns and, subsequently, between-group differences can be examined [25,26]. We included the level of self-reported SI collected at all 4 assessments (baseline, and 2, 4, and 6 weeks) and we used this information to model unique latent trajectories of SI. In general, attrition at follow-up was low: 6.8% (220/236, 2-week assessment), 10.6% (211/236, 4-week assessment), and 8.9% (215/236, 6-week assessment). We included all patients (N=236) who answered questions on SI at baseline in the LGMM and handled missing data by applying the full information maximum likelihood method [27]. We estimated a series of LGMM models with the number of classes ranging from 1 to 6. We used the fit estimates of Bayesian information criteria (BIC), Akaike information criteria (AIC), and sample size-adjusted BIC (adj.BIC) to evaluate the models. Specifically, lower values of these indexes imply a better model fit. Furthermore, we tested improvement of model fit by adding an extra class by estimating the Lo-Mendell-Rubin adjusted likelihood ratio test, the Vuong-Lo-Mendell-Rubin likelihood ratio test, and the bootstrap likelihood ratio test. We also estimated entropy, which is an estimate of classification accuracy and describes the probability of the model assigning individuals correctly in trajectories. According to the traditional approach, however, model selection is based on a combination of factors in addition to fit indices, namely parsimony of the model, theoretical justification, and interpretability [25]. In the first step, we estimated trajectories without including covariates. Second, we tested all covariates univariately for association with the most likely class membership using Pearson chi-square test (categorical variable) or analysis of variance *F* test (continuous variables). Third, we included all covariates with a univariate *P*<.2 in the 3-step approach suggested by Asparouhov and Muthén [28] to conduct multivariable testing of predictors of trajectory membership. When using this approach, covariates are not initially included in the LGMM but treated as auxiliary variables, that is, covariates do not influence the formation of classes. However, the probabilistic nature of class membership assignment is still accounted for. Thus, we first established latent classes, and then examined covariates as possible predictors of membership in identified latent classes in multivariable-adjusted analyses [28]. The analyses were carried out in Mplus version 7 (Muthén & Muthén) and in IBM SPSS version 22 (IBM Analytics).

## Results

Table 1 presents goodness of fit statistics for the LGMM analyses. We estimated models from 1 to 6 classes with fixed variance around the quadratic term and the slope, but with a freely varying intercept.

**Table 1:** Goodness of fit statistics for 1- to 6-class solutions and *P* values for test of class models.

No. of classes	Fit estimates^a^			*P* values^b^			Entropy^c^
	AIC^d^	BIC^e^	adj.BIC^f^	Vuong-Lo- Mendell-Rubin likelihood ratio test	Lo-Mendell- Rubin adjusted likelihood ratio test	Bootstrap likelihood ratio test	
1	5798	5825	5800				
2	5742	5783	5745	<.01	<.01	<.01	.69
3	5709	5764	5714	.23	.24	<.01	.77
4	5673	5742	5679	.06	.07	<.01	.85
5	5647	5730	5654	.24	.26	<.01	.86
6	5630	5727	5638	.08	.10	<.01	.87

^a^Lower values of AIC, BIC, and adj.BIC indicate better model fit.

^b^*P* values for test against model minus 1 class.

^c^Entropy estimates ranges from 0 to 1 and assess the accuracy with which models classify individuals into their most likely class; higher scores represent greater classification accuracy.

^d^Akaike information criteria.

^e^Bayesian information criteria.

^f^Sample size-adjusted BIC.

We obtained the most parsimonious model with the 4-class solution, where fit estimates (AIC, BIC, and adj.BIC) were low, the bootstrap likelihood ratio test performed with a significant *P*<0000, and both the Vuong-Lo-Mendell-Rubin likelihood ratio test and the Lo-Mendell-Rubin adjusted likelihood ratio test indicated borderline *P*=.06. Fit estimates did not decrease substantially with the addition of extra classes after the 4-class solution and, in addition, the sample size in one of the classes in the 5-class solution was very low (n=4). Further, the 5-class model performed with an insignificant Vuong-Lo-Mendell-Rubin likelihood ratio test, and the Lo-Mendell-Rubin likelihood ratio tests had *P*>.2. Based on these results, we chose the 4-class over the 5-class model. In addition, the 4-class solution had high entropy (.85) and was clinically plausible, so we settled on this model as the optimal representation of SI in our data.

**Figure 2:**
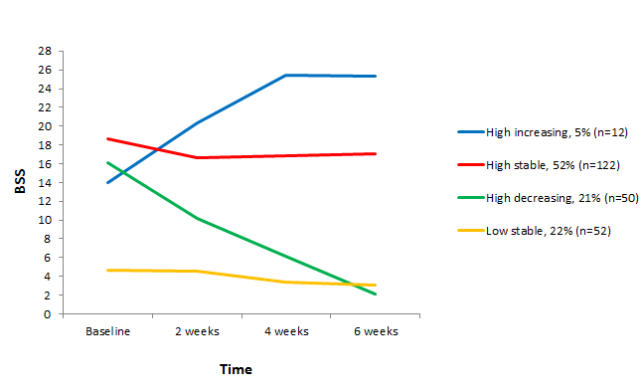
Trajectories of suicidal ideation as assessed by Beck Scale for Suicide Ideation (BSS) scores.

Of the 4 identified trajectories, 3 had high intercepts (BSS>14), indicating that individuals in these latent classes had high intensity or frequency of SI at baseline (see Figure 2). These 3 classes then developed into different patterns. The largest class, named high stable, represented 51.7% (122/236) of the participants and was characterized by a constant high score on the BSS throughout the 6-week study period. The second class, high decreasing, included 21.2% (50/236) of the sample and consisted of people with a high baseline SI score (BSS=16), which gradually decreased to an average BSS score of 2.1 at the final follow-up at week 6. Participants in the third class, high increasing, also had a high BSS score of 14 at baseline, followed by an increase throughout the study period to the highest level of SI at 6 weeks. This class consisted of 5.1% (12/236) of the participants. The fourth, low stable, consisted of 22.0% (52/236) of the participants and differed from the other 3 classes by having a considerably lower average level of SI at baseline (BSS=4.7). This level of SI was constantly low through the final assessment.

Table 2 presents characteristics by trajectory membership and univariate *P* tests of differences between them. Random allocation group, partner status, a history of attempted suicide, and higher hopelessness and depression scores indicated significant between-trajectory differences. Along with status of employment, which was nearly significant, we introduced all these variables into the multivariate predictor analyses of trajectory membership presented in Table 3 and Table 4. Participants who had received the intervention (odds ratio, OR 3.15, 95% CI 1.19–7.67) or lived with a partner (OR 3.12, 95% CI 1.27–7.67) had a 3-fold higher likelihood of membership in the high decreasing class than of membership in the high stable trajectory (see Table 3). Furthermore, relative to the high stable class, a history of attempted suicide predicted membership in the high decreasing class (OR 2.72, 95% CI 1.10–6.70). Similarly, members of the high stable class differed from members of the low stable class, who had a significantly lower level of hopelessness (OR 0.78, 95% CI 0.67–0.91). When we used the low stable class as the reference group (see Table 4), we found that participants receiving Web-based self-help (OR 3.84, 95% CI 1.22–12.12), those with a history of attempted suicide (OR 3.68, 95% CI 1.05–13.0), and those with higher levels of hopelessness (OR 1.24, 95% CI 1.01–1.52) were significantly more likely to be members of the high decreasing class. We found no significant differences in characteristics between members of the high increasing class and the high decreasing class, and therefore Table 3 or Table 4 do not display predictors between those 2 classes.

**Table 2:** Baseline characteristics of members in each suicidal ideation trajectory.

Characteristics		Latent trajectory^a^				*P* value
		High decreasing (n=46)	High increasing (n=11)	High stable (n=128)	Low stable (n=51)	
**Age in years, mean (SD)**		41 (12.3)	36 (12.0)	41 (14.3)	41 (13.8)	.66^b^
**Sex, n (%)**						.96^c^
	Women	30 (65)	8 (73)	85 (66)	33 (65)	
	Men	16 (35)	3 (27)	43 (34)	18 (35)	
**Having children, n (%)**						.58^c^
	No	27 (60)	9 (82)	78 (61)	31 (63)	
	Yes	18 (40)	2 (18)	49 (39)	18 (37)	
**Random allocation, n (%)**						.04^c^
	Control group	15 (33)	6 (55)	68 (53)	31 (61)	
	Intervention group	31 (67)	5 (45)	60 (47)	20 (39)	
**Receiving other help, n (%)**						.25^c^
	No	21 (47)	2 (18)	52 (42)	25 (50)	
	Yes	24 (53)	9 (82)	73 (58)	25 (50)	
**Paid employment, n (%)**						.07^c^
	No	19 (42)	9 (82)	67 (53)	21 (43)	
	Yes	26 (58)	2 (18)	60 (47)	28 (57)	
**Living with partner, n (%)**						.03^c^
	No	20 (44)	9 (82)	88 (65)	29 (57)	
	Yes	26 (57)	2 (18)	45 (35)	22 (43)	
**History of attempted suicide, n (%)**						<.01^c^
	No	21 (47)	2 (18)	78 (61)	36 (74)	
	Yes	24 (53)	9 (82)	49 (39)	13 (27)	
**Clinical scale, mean score (SD)**						
	Beck Depression Inventory	26 (9.9)	34 (12.0)	29 (8.3)	22 (8.6)	<.01^b^
	Beck Hopelessness Scale	15 (3.4)	15 (3.4)	15 (3.5)	12 (3.6)	<.01^b^

^a^Numbers of members in each class diverge slightly from those in Figure 2 because, while the figure is based on posterior probabilities, classes used for post hoc analyses are based on most likely class membership.

^b^Analysis of variance *F* test.

^c^Pearson chi-square test.

**Table 3:** Odds ratios (95% CI) from multivariable logistic regression analyses describing the association between baseline characteristics and membership in suicidal ideation trajectories using the high stable trajectory as the reference.

Characteristics		Low stable		High increasing		High decreasing
**Random allocation**						
	Control group	1		1		1
	Intervention group	0.82 (0.31–2.13)		0.66 (0.11–3.78)		3.15 (1.19–7.67)*
**Paid employment**						
	No	1		1		1
	Yes	1.19 (0.48–2.97)		0.18 (0.01–4.25)		1.30 (0.52–3.24)
**Living with partner**						
	No	1		1		1
	Yes	1.79 (0.68–4.70)		0.57 (0.08–4.08)		3.12 (1.27–7.67)*
**History of attempted suicide**						
	No	1		1		1
	Yes	0.74 (0.24–2.26)		8.64 (0.16–452.7)		2.72 (1.10–6.70)*
**Clinical scale**						
	Beck Depression Inventory	0.95 (0.89–1.01)		1.06 (0.95–1.19)		0.95 (0.90–1.01)
	Beck Hopelessness Scale	0.78 (0.67–0.91) **		0.87 (0.68–1.11)		0.96 (0.81–1.15)

* *P*<.05.

** *P*<.01.

**Table 4:** Odds ratios (95% C) from multivariable logistic regression analyses describing the association between baseline characteristics and membership in suicidal ideation trajectories using the low stable trajectory as the reference.

Characteristics		High increasing	High decreasing
**Random allocation**			
	Control group	1	1
	Intervention group	0.8 (0.12–5.25)	3.84 (1.22–12.12)*
**Paid employment**			
	No	1	1
	Yes	0.15 (0.01–3.48)	1.09 (0.36–3.31)
**Living with partner**			
	No	1	1
	Yes	0.32 (0.04–2.36)	1.74 (0.58–5.21)
**History of attempted suicide**			
	No	1	1
	Yes	11.7 (0.26–516.8)	3.68 (1.05–13.0)*
**Clinical scale**			
	Beck Depression Inventory	1.12 (0.99–1.26)	1.0 (0.93–1.08)
	Beck Hopelessness Scale	1.11 (0.86–1.45)	1.24 (1.01–1.52)*

* *P*<.05.

## Discussion

This study aimed to examine prototypical trajectories of SI in an adult population seeking Web-based treatment for suicidal thoughts. The results of the LGMM confirmed heterogeneity by identifying 4 distinct trajectories of SI in this population. A notable finding was that those experiencing a decrease in SI (members of the high decreasing class) differed in characteristics from those with a more stable level of SI (both high stable and low stable) during the study period. Those receiving the Web-based intervention (vs the control group) and having a history of attempted suicide were more than 3 times as likely to be members of the high decreasing class as to be members of both stable classes. Members of the high decreasing class were also more likely than members of the high stable class to be living with a partner, and they had a higher level of hopelessness at baseline than did members of the low stable class. Overall, the findings of this study yield important and new information about developmental patterns of SI and especially how these patterns associate with the Web-based self-help intervention.

### Trajectories of SI

This study has demonstrated that patterns of SI vary greatly, which is not detected using traditional analyses. While traditional analyses showed an overall small significant mean effect size in SI (Figure 1), the LGMM revealed that this effect was minimal for 74% of participants (low stable plus high stable classes), large for 21% of participants (high decreasing) who experienced a great decrease in SI, and reversed for 5% of participants who developed higher levels of SI. Because SI is an important risk factor for suicidal behaviour, it is encouraging to find that SI decreased from a worrisomely high, from a clinician’s standpoint, baseline level (BSS=16) to a considerably lower level (BSS=2) in one-fifth of participants, and that being in the intervention group was a significant predictor for membership in this class. Less reassuring is that 74% (high stable plus low stable classes) kept a stable level of SI, though it can be argued that the 22% in the low stable class had only limited potential to decrease their levels of SI. Still, the high stable class represents the majority of participants, indicating that SI persists in many despite over half in this class reporting receiving care from a professional at baseline. This indicates that SI persists (at least over a 6-week period) in many in spite of getting professional help. Although studies examining heterogeneity generally identify at least one trajectory of stable high SI, this class usually includes between 6% and 20% of participants, with one study reporting as many as 40% of participants falling into that class [9-16]. Our uncommonly large percentage of the sample with high ongoing SI may be explained by the short 6-week period compared with periods of a year or more in most other studies [10,11,13-16]. Another reason for this could be that our sample consisted of people who were required to have some level of SI at baseline, whereas the other studies examined heterogeneity in SI in samples of teenagers or psychiatric patients whether or not they reported SI [11-16]. Furthermore, a variety of instruments to measure SI was used across studies examining trajectories in SI, making comparison more challenging. Finally, the high percentage of participants in our high stable class may be illustrative of a group of chronically suicidal people who are desperate for help but are not getting what they need in the current health care system (nor in the current self-help intervention). More research will be needed to study this group and their needs.

### Characteristics of SI Trajectory Class Members

We also identified predictors of membership in trajectories. Most important, membership in the high decreasing trajectory, compared with the 2 stable trajectories, was significantly associated with having received the Web-based intervention program, indicating that the program had a reducing effect on SI. This is consistent with earlier publications on this sample, which showed a small but significant overall mean effect size (a reduction of 4.6 points on the BSS) [7,29], but adds to this knowledge by showing that the Web-based program seems to have a larger effect (a reduction of 14 points on the BSS), but in a smaller subsample. Other factors that predicted membership in this class included previous suicidal behavior, living with a partner, and hopelessness. While this is speculative, people with a history of suicidal behavior may be more motivated to change their life situation and consequently would benefit more from a Web-based self-help program aimed at reducing SI. Furthermore, regarding motivation, living with a partner was also significantly more likely in the high decreasing class than in the high stable class, and having a partner may provide emotional support and help motivate their partner with SI to improve in mental well-being and health. Finally, having feelings of hopelessness is a well-known risk factor for suicidality, and the fact that members of the high decreasing class scored significantly lower on baseline hopelessness than those in the high stable trajectory is consistent with the existing literature on this [30].

### Strength and Weaknesses

The high follow-up response rate strengthens this study, as this makes the estimation of SI trajectories more precise. Furthermore, we measured SI with a validated scale (BSS), as opposed to a single-item question on SI, which was applied in some of the other studies of heterogeneity in SI [9,11,15]. An obvious methodological concern is the low power of this study, with only 236 participants. This is especially a problematic issue when 1 trajectory (the high increasing) had very few members, as the power to predict membership in that class is low. Had the sample been larger, we would have had the power to detect predictors of membership in the high increasing class, which obviously is an important group of people to characterize and monitor, as their level of SI increased during the study period. Table 2 shows that most participants in the high increasing trajectory were already receiving other help, had attempted suicide before, had higher BDI scores, and were single or unemployed. Hence, this group of participants was characterized by known risk factors of suicidality. Based on this and because less than half of the members of this trajectory had received the intervention, we have reasons to believe that the worsening course of SI in this group probably does not reflect an iatrogenic effect of the Web-based self-help.

### Conclusion

The analyses of trajectories of SI have provided clinically interesting information. In particular, it was notable that being assigned to the Web-based self-help program, having a history of suicidal behavior, having a partner, and having lower baseline levels of hopelessness were associated with experiencing a large decrease in SI during the 6-week study period, even though this applied to a small proportion of the sample. A discouraging finding was that a large proportion of the Web-based help seekers experienced persistently high levels of SI, and future research should address whether this group might benefit more from tailored Web-based programs or from face-to-face help. Recently, this Web-based self-help program to reduce SI was found to be cost effective too [29], so it is a promising bid for an intervention that can be offered to many via Web-based technology, although future studies are still needed to confirm a long-term effect of the program on SI.

## Abbreviations

adj.BIC: sample size-adjusted Bayesian information criteria
AIC: Akaike information criteria
BDI: Beck Depression Inventory
BIC: Bayesian information criteria
BSS: Beck Scale for Suicide Ideation
LGMM: latent growth mixture modelling
OR: odds ratio
SI: suicidal ideation
